# Sleep Disturbances in MCI and AD: Neuroinflammation as a Possible Mediating Pathway

**DOI:** 10.3389/fnagi.2020.00069

**Published:** 2020-05-08

**Authors:** Victoria M. Pak, S.-Hakki Onen, Donald L. Bliwise, Nancy G. Kutner, Katherine L. Russell, Fannie Onen

**Affiliations:** ^1^Nell Hodgson Woodruff School of Nursing, Emory University, Atlanta, GA, United States; ^2^Centre de Sommeil, Hôpital de la Croix-Rousse, Lyon, France; ^3^INSERM U128, Université de Lyon, Lyon, France; ^4^Department of Neurology, Emory University, Atlanta, GA, United States; ^5^Department of Rehabilitation Medicine, Emory University, Atlanta, GA, United States; ^6^CHU Bichat–Claude-Bernard, AP-HP, Service de Gériatrie, Paris, France; ^7^CESP & INSERM 1178 Université Paris Sud, Paris, France

**Keywords:** neuroinflammation, sleep, mild cognitive impairment, Alzheimer’s disease, aging

## Abstract

Mild cognitive impairment (MCI) and Alzheimer’s disease (AD) affect a high proportion of the elderly population with an increasing prevalence. Sleep disturbances are frequent in those with MCI and AD. This review summarizes existing research on sleep disturbances and neuroinflammation in MCI and AD. Although strong evidence supports various pathways linking sleep and AD pathology, the temporal direction of this central relationship is not yet known. Improved understanding of sleep disturbance and neuroinflammation in MCI and AD may aid in the identification of targets for their prevention.

## Introduction

Alzheimer’s disease (AD) is a leading public health problem (Ballard et al., 2011) affecting 24 million people worldwide (Ballard et al., 2011; Reitz and Mayeux, 2014). Descriptive evidence implies that sleep may play a crucial role in optimal daytime cognitive functioning, attention, executive functioning, and memory (Diekelmann and Born, 2010; Killgore, 2010). Disturbed sleep is associated with cognitive disorders (da Silva, 2015), and early observational data from institutionalized AD patients showed worse nocturnal sleep among those with more severe dementia (Bliwise et al., 1995). Epidemiological research among older adults has shown that both short and long sleepers have worse cognitive function (Potvin et al., 2012; Devore et al., 2014) and greater cognitive decline in contrast to adults sleeping 7 or 8 h per night (Keage et al., 2012; Benito-León et al., 2013). Thus, a U-shaped association between sleep duration and cognitive outcomes has been suggested (Yaffe et al., 2014). Both cross-sectional (Muangpaisan et al., 2008; Naismith et al., 2010; Guarnieri et al., 2012) and longitudinal studies (Westerberg et al., 2012), have shown that sleep disturbance is linked to mild cognitive impairment (MCI) and AD.

Patients with dementias, especially AD, often have disrupted nocturnal sleep. When monitored in overnight sleep studies (Vitiello et al., 1990; Montplaisir et al., 1998), they have earlier bedtimes and much more variable wake-up times than those without AD (Bliwise et al., 1992). Approximately 25–44% of patients with AD reported having sleep disturbances, although sleep was measured via self-report (Guarnieri et al., 2012). Nocturnal sleep disturbance is more frequent in dementia patients with Lewy bodies than in patients with AD, although nocturnal sleep disturbances in AD are more associated with advanced disease (Bliwise et al., 2011). There may also be a higher prevalence of sleep apnea in AD patients, though this remains a controversial area (Bliwise, 1996; Bliwise et al., 2019). Nevertheless, a potential causal role for sleep apnea in impaired cognition in the aged could be exacerbated by sleep fragmentation, inflammation, oxidative stress, and hypoxemia, all of which are features of disturbed respiration during sleep (Daulatzai, 2012, 2013). In this review, we examine relevant existing human research on neuroinflammation and sleep disturbance in MCI and AD.

## Importance of Exploring Microglia, Neuroinflammation, and Sleep in MCI and AD

We propose a model in which disturbed sleep may play a crucial causal role for cognitive decline in old age. This places neuroinflammation as the most important mediating variable underlying many of the associations noted to date. Sleep disturbance may be a major risk factor for AD and is associated with increased inflammation (Hansson et al., 2006; Alvarez et al., 2007). The amyloid cascade hypothesis proposed by Hardy and Higgins (1992) postulates that the accumulation of insoluble amyloid β (Aβ) is the primary driver of AD pathology. However, research has demonstrated neuroinflammatory responses connected to Aβ deposition common to both sleep disturbance and AD.

Microglia are a type of glial cell that is common in the central nervous system (CNS) and functions as a primary line of immune defense (Nayak et al., 2014). They manage immunosurveillance and mediate inflammation, both of which are suggested to be important in AD (Olsson et al., 2013). Activated microglia appear to have a beneficial effect in response to brain injury, although uncontrolled microglial activation that is part of aging may lead to neuronal dysfunction and cognitive decline (Ojo et al., 2015). Upon exposure to Aβ, microglia and astrocytes release cytokines, interleukins, nitric oxide, and other potentially cytotoxic molecules (McGeer and McGeer, 2010; Krabbe et al., 2013; Heneka et al., 2015). Twenty-four-hour sleep deprivation followed by 24-h recovery was associated with elevated levels of IL-6 and microglial activation in the mouse hippocampus, although not in the cortex (Zhu et al., 2012). Chronic sleep restriction and low-level microglial activation may promote neuroinflammation and increase the brain’s vulnerability to insult (Atienza et al., 2018). Xie et al. (2019) have shown that chronic sleep fragmentation can induce dysfunction of intracellular protein degradation and activate microglia predominately in the hippocampus, which in turn may induce this pro-inflammatory response (Xie et al., 2019). Rodent models of chronic sleep deprivation have reported microglial morphological changes, including enlargement of the cell body and increased expression of pro-inflammatory cytokines (Hsu et al., 2003; Wisor et al., 2011). Thus, microglial dysfunction and neuroinflammation may contribute to the pathophysiology of sleep disorders.

Additionally, intrinsic molecular clock control within microglia regulates sleep–wake cycle-dependent changes in synaptic strengths (Hayashi et al., 2013). This intrinsic circadian control within microglia is an example of a peripheral clock of the circadian sleep-wake system. These are centrally directed from the suprachiasmatic nucleus (SCN), which controls metabolomic changes and synchronizes tissues within the body in response to internal and external “zeitgebers” such as light (Musiek, 2017). These cellular circadian rhythms are often expressed through a transcriptional–translational feedback loop (TTFL) in which the transcriptional factors CLOCK and BMAL1 heterodimerize and bind to E-box sequences in target gene promoters to drive rhythmic expression of Period (Per1-3) and Cryptochrome (Cry1-2) proteins. These Per and Cry proteins then form a complex to return to the nucleus and inhibit CLOCK- and BMAL1-mediated gene expression, completing the feedback loop (Eckel-Mahan and Sassone-Corsi, 2013; Gachon et al., 2017). Studies in mice show that when *Bmal1* or *Clock* is deleted or deficient, they produce arrhythmic circadian cycles in the SCN (Eckel-Mahan and Sassone-Corsi, 2013; Krishnaiah et al., 2017). This process may be disrupted in AD patients, as differences in the phase of these clock gene rhythms have been observed in the brains of individuals with AD when compared to controls (Cermakian et al., 2011; Weissova et al., 2016). Additionally, Ni et al. (2019) show that reduced expression of BMAL1 (a clock gene) is responsible for increased expression of an inflammatory type of microglia in APP knockout mice (Ni et al., 2019). This circadian rhythm dysfunction links AD and sleep disturbance pathology (Musiek, 2017).

Recent research has demonstrated the importance of a missense mutation in the triggering receptor expressed on myeloid cells 2 (TREM2) in increasing one’s risk for AD (Guerreiro et al., 2013; Jonsson et al., 2013; Wang et al., 2015). The R47H variant of the gene confers a 2–4.5-fold increased risk of developing late-onset AD. Behind the APOE ε4 gene, these results place *Trem2* as the strongest associated risk gene for AD (Guerreiro et al., 2013; Jonsson et al., 2013). In studies of mice with Aβ accumulation, deletion of *Trem2* significantly reduced the number of plaque-associated macrophages (Jay et al., 2015). Deletion of *Trem2* also led to decreases in astrocytosis and declines in inflammatory cytokines IL-1β, IL-6, and TNF-α (Jay et al., 2017). Additionally, studies have shown that microglia and macrophages lacking TREM2 have a reduced ability to clear Aβ and apoptotic cells from the brain (Savage et al., 2015). In addition to TREM2, research has explored other microglia-specific receptors and their role in immune response in AD. CX3CL1 is an inhibitory chemokine that binds to CX3CR1 on the surface of microglia, regulating microglial activity and migration in injury conditions (Limatola and Ransohoff, 2014). CX3CL1 may also dose-dependently reduce expression of nitric oxide, IL-6, and TNF-α, and suppress neuronal death induced by microglial activation. Disrupting the receptor can lead to system inflammation and microglial neurotoxicity (Jung et al., 2000; Zujovic et al., 2000; Mizuno et al., 2003; Cardona et al., 2006). Plasma levels of CX3CL1 have been shown to be elevated in patients with AD and more so in patients with MCI, possibly due to increased inflammation preceding AD pathology (Kim et al., 2008). Additionally, postmortem analysis shows significantly lower levels of CX3CL1 in the hippocampus of AD brains compared to controls without dementia (Cho et al., 2011). Microglia, particularly those that have been activated, exhibit GABA_*B*_ receptors (Kuhn et al., 2004). This is important as GABA decreases the release of pro-inflammatory cytokines IL-6 and TNF-α by activating astrocytes and microglia through this receptor (Lee et al., 2011).

Astrocytes reside in the extracellular space (ECS) of the CNS and are also involved in the clearance of metabolites from the brain. They are responsible for clearing Aβ from parenchyma through the recently described glymphatic system (Jessen et al., 2015). There is a 60% increase in the ECS during sleep, allowing increased flow of interstitial fluid from para-arterial to para-venous space that doubles the rate of Aβ clearance at night (Cedernaes et al., 2017; Mander et al., 2016). In order for this to occur, low adrenergic input is required to the area. The locus coeruleus (LC), best known for its role in alertness and attention and as a center of noradrenergic neurons, acts as the main driver of metabolite clearance in the brain by reducing its adrenergic input (Mander et al., 2016). Any activation of the LC during the night, which occurs in obstructive sleep apnea (OSA), may affect its role in sleep-mediated memory consolidation (Twigg et al., 2010; Rosenzweig et al., 2016). Conversely, normal activation of adrenergic neurons in the LC during the day is neuroprotective, as noradrenaline (NA) is anti-inflammatory and anti-oxidative (Heneka et al., 2015). NA is also responsible for reducing TNF-α expression in microglia, interferon γ-induced expression of class II antigens in astrocytes, and cytokine induction of type 2 nitric oxide synthase expression in astrocytes and microglia (Feinstein et al., 2016). The number of neurons in the LC naturally declines with age, but this occurs more significantly in individuals with AD (Robinson, 1975; Heneka et al., 2015). In fact, this reduction in LC neurons is significantly correlated with the number of plaques, the number of neurofibrillary tangles, and the severity of dementia (Bondareff et al., 1981, 1987). It is hypothesized that increased damage to the LC in AD pathology results from hyperphosphorylated tau, which appears before tau lesions or Aβ accumulation in the cortex (Braak et al., 2011).

Melatonin, a major hormone controlling circadian rhythm and the sleep–wake cycle, is decreased significantly in the cerebrospinal fluid (CSF) and blood of individuals with AD (Zhou et al., 2003; Wu and Swaab, 2005). Additionally, reductions of MT2, a melatonin receptor, are found in the hippocampus of AD patients (Savaskan et al., 2005, 2002). In addition to its role in the sleep–wake cycle, melatonin inhibits tau phosphorylation (Deng et al., 2005) and reduces the release of APP from neurons (Lahiri, 1999), both hallmarks of AD. Melatonin also plays an anti-inflammatory role by inhibiting the activation of nuclear factor kappa B (NF-κB) (Tamura et al., 2009) and reducing adherence of leukocytes and neutrophils to endothelium, preventing vascular permeability (Lotufo et al., 2006). Melatonin is released from the pineal gland, which can be reduced in volume or undergo calcification in AD (Bumb et al., 2013, 2014). When Aβ interacts with toll-like receptors in the pineal gland, subsequent release of pro-inflammatory factors inhibit synthesis of melatonin through the NF-κB pathway (Cecon et al., 2015). These pineal gland volume and function changes in AD are also significantly associated with sleep disturbances and insomnia (Matsuoka et al., 2017).

TNF-α plays an important role as a pro-inflammatory cytokine and is elevated in the brains and plasma of individuals with AD (Chang et al., 2017). TNF-α binds to TNFR1 or TNFR2 to activate a cytokine cascade. Overexpression of TNFR1 in the hippocampi of mice resulted in activation of Aβ-induced apoptosis, while mice lacking the receptor exhibited reduced microglial activation and improved cognition performance (He et al., 2007; Li et al., 2004). As noted above, microglia produce TNF-α in response to Aβ. Perpetuating this cycle, TNF-α can then promote Aβ production through upregulation of β-secretase (Liao et al., 2004). IL-1β is another pro-inflammatory cytokine involved in Aβ plaque development that is found in increased levels in the hippocampus and prefrontal cortex of individuals with AD (Cacabelos et al., 1994). IL-6 is a cytokine whose overproduction can lead to neuroinflammation (Rothaug et al., 2016). Elevated levels in midlife predict cognitive decline 10 years later and are elevated in the CSF and plasma of individuals with AD (Blum-Degena et al., 1995; Singh-Manoux et al., 2014). In addition to its release by glial cells in response to Aβ, activation of IL-6 receptors enhance APP transcription and expression (Chong, 1997; Ringheim et al., 1998). Elevated levels of these pro-inflammatory cytokines have also been linked to sleep disturbances, such as excessive daytime sleepiness (EDS), obstructive sleep apnea, and narcolepsy (Vgontzas et al., 1997; Okun et al., 2004). Studies simulating acute sleep loss in humans have shown TNF-α, IL-6, and cellular adhesion molecules to be dysregulated (Vgontzas et al., 2004; Frey et al., 2007; Chennaoui et al., 2011). Even modest sleep restriction is associated with secretion of IL-6 in both men and women and TNF-α in men (Mullington et al., 2016).

It is believed that impaired permeability of the blood–brain barrier (BBB) may be related to neuroinflammation and sleep disruption (Atienza et al., 2018). Specifically, chronic restriction of either total sleep time (He et al., 2014) or REM sleep results in a reduction in BBB integrity (Montagne et al., 2015). Sleep fragmentation may lead to age-related disruption of the BBB’s ability to block proinflammatory compounds (Atienza et al., 2018). Increased BBB permeability in aging may augment low-grade inflammation on cerebral integrity and mediate the relationship between disrupted sleep, dysfunctional adiposity, and impaired cognition (Atienza et al., 2018). These results suggest that disturbed sleep may play a causative role in neuroinflammation and subsequent cognitive decline.

Existing human studies on microglia and neuroinflammation are limited in that they do not measure sleep explicitly (Schuitemaker et al., 2009; Olsson et al., 2013) but may hold relevance for possible causal pathways linking poor sleep to cognitive decline. CSF YKL-40, a glycoprotein and biomarker of glial inflammation, is associated with a cerebral structural signature distinct from that related to p-tau neurodegeneration at the earliest stages of cognitive decline due to AD (Gispert et al., 2016). In order to investigate if microglial markers could differentiate between AD and controls, and secondly between stable MCI and those progressing to AD, the Olsson et al. study quantified YKL-40 and sCD14 in CSF from 96 AD patients, 65 healthy controls, and 170 patients with MCI from baseline and over 5.7 years (Olsson et al., 2013). YKL-40, and not sCD14, was significantly elevated in AD compared with healthy controls (*p* = 0.003). Furthermore, according to the National Institute of Neurological and Communicative Disorders and Stroke and the Alzheimer’s Disease and Related Disorders Association (NINCDS-ADRDA) (Olsson et al., 2013), YKL-40 and sCD14 were increased in MCI patients who converted to vascular dementia (VaD) (*p* = 0.029 and *p* = 0.008), but not to AD. However, when stratified according to CSF levels of tau and Aβ42, YKL-40 was elevated in those with an AD-indicative profile compared with stable MCI with a normal profile (*p* = 0.037). In addition, YKL-40 and sCD14 were very stable in AD patients with strong correlation between time-points (*r* = 0.94, *p* = 3.4 × 10−25; *r* = 0.77, *p* = 2.0 × 10−11) and the cortical damage marker T-tau (Olsson et al., 2013). Thus, microglial markers appear stable for monitoring CNS inflammation and microglia activation in clinical trials (Olsson et al., 2013). Moreover, YKL-40 differentiates between AD and controls, between stable MCI to AD, and those that convert to AD and VaD. In order to clarify the role of disturbed sleep and its impact on neuroinflammation, it will be important to measure sleep explicitly in future studies.

Studies conducted in human subjects have demonstrated that neuroinflammation, which includes microglia activation and increases in the levels of pro-inflammatory cytokines in the brain, may lead to cognitive function decline (Schuitemaker et al., 2009). Schuitemaker et al. (2009) conducted a study examining levels of pro-inflammatory cytokines in patients with MCI and probable AD. In this study, 145 subjects had probable AD and 67 had MCI. Patients were diagnosed with MCI according to Petersen et al. (1999): failing in one or more cognitive domains or a greater cognitive decline than that expected for the person’s age or education but not interfering notably with daily activities (Petersen et al., 1999). Patients were diagnosed with probable AD according to McKhann et al. (1984): the gradual worsening of specific cognitive functions such as language, motor skills, and perception (McKhann et al., 1984). The MCI patients were further classified as having either a low-risk or high-risk for AD according to their Aβ42 and tau levels in CSF, with cut-off values from previously published work (Schoonenboom et al., 2005). High risk CSF consisted of Aβ42 ≤ 494 pg/mL and tau > 356 pg/mL and low risk CSF consisted of Aβ42 > 494 pg/mL and tau > 356 pg/mL. CSF and serum CRP levels were significantly higher in MCI compared to AD patients after adjustment for age, APOE ε4 genotype, and cardiovascular diseases. This difference remained present in patients with a low-risk biomarker profile for AD after adjustment for the abovementioned covariates. CSF IL-6 levels were also significantly higher in MCI patients with a low-risk CSF profile (Schuitemaker et al., 2009). Again, these studies have not assessed sleep *per se* but do suggest that associations between cognitive impairment and sleep noted in the literature may well be reflecting such mediational effects.

## Sleep Disturbances in Alzheimer’S

The adverse effects of sleep on the production and clearance of proteins have been seen in neurodegenerative diseases such as AD (Ju et al., 2017; Olsson et al., 2018; Shokri-Kojori et al., 2018). Sleep has a role in regulating neuronal activity that affects the release of glymphatic proteins, Aβ, and τ (Huang et al., 2012). The brain relies on the glymphatic clearance pathway to remove these waste materials (Xie et al., 2013). Studies have shown positive associations between sleep deprivation, OSA, and increased efflux of these proteins in the brain. Spira et al. (2013) found that older adults with shorter sleep duration have an increase in Aβ (Spira et al., 2013). Another study conducted with healthy volunteers revealed that a night of total sleep deprivation led to a physiological increase in cerebral Aβ42, an isoform of Aβ (Ooms et al., 2014). A 2-year prospective longitudinal study conducted in community-dwelling healthy older adults found that OSA was associated with markers of increased amyloid burden (Sharma et al., 2018). A study of OSA patients from the Korean Genome and Epidemiology Study revealed significant Pittsburgh compound B (PiB) deposition compared to controls at the right posterior cingulate gyrus and the right temporal cortex; there was no area of higher uptake in the controls compared with the OSA group (Yun et al., 2017). The study found that OSA accelerates amyloid deposition, contributing to the development or progression of AD (Yun et al., 2017).

Clarifying the underlying causes of sleep disturbances in AD can help to improve quality of life and understanding of disease progression. A prior study by Lee et al. (2007) investigating whether reports of daytime sleepiness in AD were correlated with lower functional status showed that EDS is linked to lower quality of life in AD subjects (Lee et al., 2007). EDS has been reported to range from 44.5 (Guarnieri et al., 2012) to 52.1% (Park et al., 2011) in AD patients. Despite this high prevalence, there is a lack of research on the mechanistic causes of sleepiness in AD, which will have important implications for improving quality of life and disease progression.

Recent research reveals a correlation between self-reported EDS and Aβ deposition in cognitively normal subjects (Carvalho et al., 2018; Spira et al., 2018). Spira et al. (2018) showed that EDS is associated with more than 2.5 times the odds of Aβ deposition at follow-up 15.7 years later in 124 cognitively normal subjects (Spira et al., 2018). However, they did not look at objectively measured sleepiness and did not adequately control for variables such as sleep quality, which may be linked to higher CSF Aβ (Ooms et al., 2014); sleep duration, which may influence Aβ accumulation (Spira et al., 2013); or sleep apnea, which is linked to sleepiness and may influence Aβ (Spira et al., 2014; Bu et al., 2015). Another study of 2,172 subjects found that baseline EDS is associated with a longitudinal increase in Aβ accumulation [measured by Pittsburgh compound B positron emission tomography (PiB-PET)] in elderly persons without dementia (Carvalho et al., 2018). One study of 1,041 non-demented participants over 65 years found that increased daytime sleepiness and sleep inadequacy was a risk factor for development of dementia, which occurred in 78 participants over the following 3 years (Tsapanou et al., 2015). Additionally, increased Aβ deposition in the brainstems of patients with more frequent nocturnal awakenings is associated with cognitive impairment (You et al., 2019). Prior studies have confirmed this link between poor sleep and/or sleep disturbance and cognitive impairment (Blackwell et al., 2006; Tworoger et al., 2006; Xu et al., 2011; Yaffe et al., 2011). Aβ deposition as a result of less adequate sleep, more sleep problems, and greater daytime somnolence has been reported in AD-sensitive brain regions and is associated with greater AD pathology (Sprecher et al., 2015, 2017). This relationship between sleep and Aβ accumulation has also been demonstrated in subjects with pre-clinical AD (Ju et al., 2013). A cross-sectional study of 145 cognitively normal individuals (half of which had a parental history of late-onset AD) measured sleep quality using wrist actigraphy and Aβ levels in CSF samples (Ju et al., 2013). 22% of the sample met their definition of “preclinical” AD, in which CSF Aβ42 levels were 500 pg/mL or less. This group experienced worse sleep quality and more frequent napping (Ju et al., 2013).

The apolipoprotein (APOE) ε4 allele significantly increases one’s risk for developing AD. One copy of the allele can increase risk of developing AD by nearly threefold, while two alleles may increase one’s risk by 8–15 times (Corder et al., 1993). APOE ε4 has also been linked to sleep disturbances. For example, Koo et al. (2019) showed that carriers of the gene more frequently experienced disrupted sleep as compared to non-carriers (Koo et al., 2019). Sleep quality measured by polysomnography (PSG) and wrist actigraphy was also shown to be lower in individuals with APOE ε4 as compared to non-carriers (Drogos et al., 2016). Additionally, APOE ε4 has been linked to an increased risk of developing sleep apnea (Kadotani et al., 2001; Gottlieb et al., 2004).

## Sleep Disturbances in Mci

Sleep disturbances are not restricted to those with AD but are also prevalent in patients with MCI (da Silva, 2015). MCI is viewed as a transitional stage between normal decline of cognitive aging to dementia, which is a general term for loss of memory and other cognitive abilities severe enough to interfere with daily life (Muangpaisan et al., 2008). The specific transition between normal aging and MCI can be subtle and the distinction between MCI and very early dementia is challenging (da Silva, 2015). Patients affected with this condition have a higher conversion rate to AD, with an estimated average rate of 10–15% annually (Muangpaisan et al., 2008). MCI patients may show intermediate levels of electroencephalographically (EEG) defined sleep instability relative to AD patients (Maestri et al., 2015). Sleep disturbance is prevalent and may be predictive of cognitive decline in older adults and in those with neurodegenerative disorders (da Silva, 2015). Though these studies suggest linkage between sleep disturbance and MCI, the underlying mechanisms of sleep disturbances remain unclear.

A prior small case-control study of 16 older adults and 8 MCI subjects found that amnesiac MCI (aMCI) patients had lower delta and theta power during non-REM sleep and spent less time in slow-wave sleep. This is concerning since the sleep changes common in aMCI may interfere with sleep-dependent memory consolidation (Westerberg et al., 2012). Brayet et al. (2016) reported slower EEG frequencies during REM, particularly for amnestic- relative to non-amnestic MCI (Brayet et al., 2016). Additionally, at least one study has indicated that the associations between disrupted sleep and impaired cognition in MCI may be, at least in part, moderated by APOE genotype (Hita-Yanez et al., 2012).

## Integration of Evidence: Neuroinflammation as a Mediator of the Association of Sleep Disturbance With AD

**FIGURE 1:**
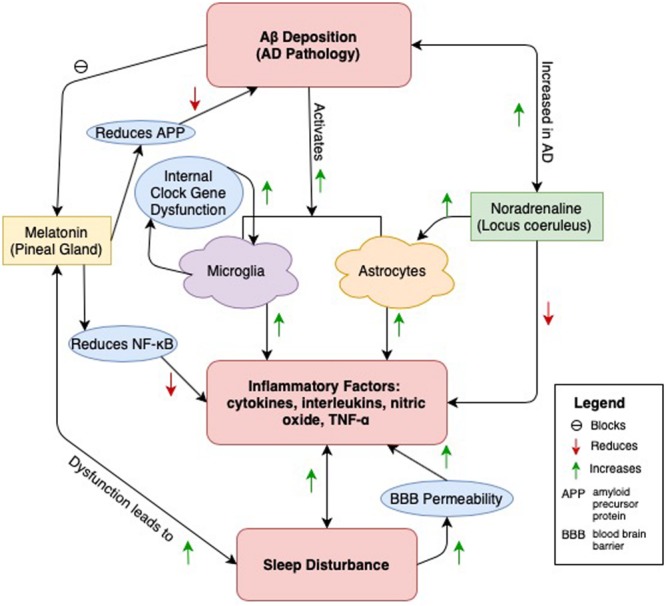
A central pathway through which sleep disturbance and AD pathology is connected is through neuroinflammation caused by Aβ deposition (Heneka et al., 2015). Microglia and astrocytes are both activated and release inflammatory factors in response to Aβ (Krabbe et al., 2013). Additionally, disruption of internal clock gene function in microglia causes an increase in their release (Ni et al., 2019). AD pathology may damage neural pathways and lead to calcification within the pineal gland (Bumb et al., 2013, 2014). This leads to a reduction in the amount of melatonin which in turn causes sleep disturbance (Matsuoka et al., 2017). This relationship is bidirectional, as sleep disturbance is linked to disruptions of melatonin release (Wu and Swaab, 2005). Melatonin has anti-inflammatory properties, reducing the release of these factors by attenuating the release of NF-κB (Cecon et al., 2015). Additionally, melatonin reduces amyloid precursor protein (APP), thus protecting against Aβ creation (Lahiri, 1999). However, Aβ, by binding toll-like receptors of the pineal gland, may block melatonin release, leading to sleep disturbance in AD (Cecon et al., 2015). Noradrenaline, released from the locus coeruleus (LC) also plays an anti-inflammatory role, as the neurotransmitter can reduce the release of inflammatory factors (Feinstein et al., 2016). Low adrenergic input from the LC to the extracellular space (ECS, location of astrocytes) during sleep is required to allow Aβ and other metabolite clearance to occur (Mander et al., 2016). The LC-noradrenergic system activates with cortico-hippocampal neuronal replay during NREM EEG slow oscillations, suggesting a prominent role in sleep dependent memory consolidation (Rosenzweig et al., 2016; Twigg et al., 2010). Thus, inappropriate activation of the LC may compromise its role in memory consolidation during sleep and reduce Aβ clearance, leading to AD pathology. Finally, sleep disturbance has been independently linked to the release of inflammatory factors and increased blood brain barrier (BBB) permeability (He et al., 2014; Montagne et al., 2015). Sleep disturbance is correlated with increased Aβ deposition and may be occurring through any of the above pathways (Ju et al., 2017; Ooms et al., 2014; Spira et al., 2013).

We propose neuroinflammation as the mediator between AD and sleep disturbance. AD and sleep disturbance, as described in Figure 1. Evidence has accumulated linking sleep disturbance to Aβ deposits in the brain, particularly in regions that Aβ is typically found in AD. Aβ also serves as a target of inflammatory response, attracting microglia that release cytokines, interleukins, and other potentially cytotoxic molecules. Other pathological changes in melatonin and NA release, both serving roles in sleep-wake neural circuitry and functioning in anti-inflammatory responses, reduce the brain’s ability to respond to inflammation generated by Aβ deposition. Sleep disturbances also independently promote a rise in inflammatory cytokines and interleukins and are separately linked to cognitive decline and AD. Fewer studies, however, have examined all three outcomes – AD, sleep disturbance, and inflammatory markers – within a human population. Most have addressed only one or two of these relationships at a time.

Two studies have examined all three outcomes within mouse models. Ni et al. (2019) studied APP knock-in (APP-KI) and wild-type (WT) mice injected with an agonist for REV-ERB designed to reduce expression of *BMAL1*, a central clock gene (Ni et al., 2019). This disruption of CLOCK/BMAL1-driven transcriptional loops impaired microglia in APP-KI mice. This led to NF-κB activation, increasing expression of pro-inflammatory genes (TNF-α, IL-1β, and IL-6) (Ni et al., 2019). This study demonstrates that clock gene disturbance in microglia is involved in early onset of AD by inducing chronic neuroinflammation (Ni et al., 2019). Thus, disturbed sleep and its subsequent triggering of inflammatory responses may play a critical role in AD pathology development. Cecon et al. (2015) also studied these outcomes in a mouse model. They treated rat pineal glands with Aβ_1__–__40_ or Aβ_1__–__42_ before inducing melatonin synthesis with NA (Cecon et al., 2015). They found that Aβ interfered with melatonin binding. Additionally, 52 inflammatory genes were upregulated in response to Aβ. Through the NF-κB pathway, these inflammatory factors inhibited synthesis of melatonin, reducing production of the hormone by 75% (Cecon et al., 2015). This study demonstrates a way in which Aβ, a hallmark of AD, may interfere with sleep regulation through disruption of melatonin via inflammatory pathways.

We have included a summary of the key human and animal studies discussed above in Table 1. It is unclear whether sleep disturbance leads to AD through inflammation or whether AD pathology leads to inflammation and subsequent sleep disturbance. The amyloid cascade hypothesis has provided a platform for researchers to target Aβ in hopes of developing effective AD drugs. Given that Aβ deposition triggers inflammation in the brain, it is hypothesized that treatment of Aβ would solve both problems. However, clinical trials in this area have been lacking. Others have instead focused on the brain’s inflammatory response to Aβ, specifically whether non-steroidal anti-inflammatory drugs (NSAIDs) may reduce AD risk or stop progression of the disease. These studies have also been inconclusive (Miguel-Álvarez et al., 2015). Largely missing from the literature are human studies examining all three outcomes: AD pathology, sleep disturbance, and inflammatory markers. Additionally, randomized studies examining the effect of treating sleep disturbance on AD risk would aid in determining the temporal relationship between AD and sleep (Irwin and Vitiello, 2019).

**TABLE 1:** Key studies examining AD, MCI, sleep disturbance and/or inflammation.

**Title**	**Author**	**Methods**	**Results**	**Conclusion**
**Human studies (AD)**				
Microglial markers are elevated in the prodromal phase of Alzheimer’s disease and vascular dementia	Olsson et al., 2013	CSF measured from 96 AD patients, 65 healthy controls, and 170 patients with MCI from baseline and over 5.7 years.	When stratified according to CSF levels of tau and Aβ42, YKL-40 was elevated in those with an AD-indicative profile compared with stable MCI with a normal profile. YKL-40 was significantly elevated in AD subjects and both YKL-40 and sCD14 were increased in MCI patients who converted to vascular dementia.	Biomarker of glial inflammation may be used to differentiate between AD patients, controls, and MCI. Supports microglial inflammation in AD.
Excessive daytime sleepiness and napping in cognitively normal adults: associations with subsequent amyloid deposition measured by PiB PET	Spira et al., 2018	One hundred and twenty-eight participants of the Baltimore Longitudinal Study of Aging Neuroimaging Substudy completed subjective excessive daytime sleepiness (EDS) questionnaire, PiB PET scan and MRI. Analysis focused on mean cortical DVR (index of global amyloid deposition) in the frontal, cingulate, lateral temporal, parietal, and lateral occipital regions of the brain.	Patients with EDS had more than 2.5 times the odds of having Aβ deposition an average of 15.7 years later.	Sleepiness may be related to AD pathology and contribute to Aβ deposition.
**Human studies (MCI)**				
Inflammatory markers in AD and MCI patients with different biomarker profiles	Schuitemaker et al., 2009	Plasma serum and CSF levels of IL-6, ACT, CRP, Aβ42, p-tau, and total tau taken from 145 patients with probable AD and 67 with MCI. High to low risk MCI was established using Aβ42/tau profile.	CSF and plasma CRP levels higher in MCI patients (*p* < 0.01), even in those with a low-risk biomarker profile. CSF IL-6 levels higher in MCI patients with low-risk profile.	Inflammation may be involved in AD/cognitive decline before Aβ and tau pathology appears.
**Animal studies**				
Amyloid β peptide directly impairs pineal gland melatonin synthesis and melatonin receptor signaling through the ERK pathway	Cecon et al., 2015	Adult male Wistar rats were kept under 12 h light, 12 h dark cycle. Pineal glands were harvested and then treated with Aβ_1__–__40_ or Aβ_1__–__42_ before stimulation of melatonin synthesis with noradrenaline. HEK293 cells were transfected to express melatonin receptors MT1 and MT2. After Aβ incubation, glands were processed to nuclear protein extraction and supershift assay was performed by incubating protein extracts. TNF content was measured by ELISA kits. Real-time RT-PCR, radioligand binding experiments, microplate BRET assay, and SDS-page were conducted.	Glands incubated with Aβ_1__–__40_ (75% reduction) or Aβ_1__–__42_ (40% reduction) showed impaired noradrenaline-induced NAS and melatonin production. Aanat mRNA levels were reduced in those treated with Aβ_1__–__40_. Those treated with Aβ also showed dose-dependent increases in NF-κB. Fifty-two inflammatory genes related to the TLR family, TLR adaptors, MAP kinases of the JNK family, and most cytokine-related genes were upregulated in treated glands. TNF release peaked in response to Aβ. Melatonin receptor MT1 binding sites were reduced by 40% in HEK293 cells treated with Aβ.	Aβ interferes with melatonin binding and signaling and stimulates inflammation in the pineal gland.
An impaired intrinsic microglial clock system induces neuroinflammatory alterations in the early stage of amyloid precursor protein knock-in mouse brain	Ni et al., 2019	Inflammatory and clock genes examined in microglia isolated from 2 month old male amyloid precursor protein knock-in (APP-KI) and wild-type (WT) mice using CAGE deep sequencing and PCR. Mice were kept in dark-light conditions where Zeitgeber time 0 (ZT0) was lights on and ZT12 was lights off. All mice were administered SR9009 (synthetic agonist for REV-ERB) for 14 days. Brain cortical tissues collected from APP-KI mice with and without SR9009 injection. mRNA from isolated microglia of each group at different time points subjected to RT-PCR. Locomotor activity was tested after 14 days of treatment and novel object recognition and Y-maze tests were conducted.	Expression of cortical clock genes occurred at different time points in APP-KI mice, suggesting impaired transcriptional feedback loops. mRNA expression of inflammatory genes were significantly higher in cortical microglia of APP-KI mice, particularly during ZT14. Expression of TNF-α, IL-1β, and IL-6 were significantly higher during the day in APP-KI mice. A significant increase in REV-ERBα mRNA was detected in APP-KI after treatment with SR9009. Aβ deposition were observed in hippocampus and cerebral cortex of 6 month old, but not 2 month old, APP-KI mice, but treatment with SR9009 increased expression in 2 month old APP-KI.	Expression of clock genes and pro-inflammatory genes (TNF-α, IL-1β, and IL-6) increased in microglia of APP-KI mice. Clock gene disturbance in microglia is involved in onset of AD pathology through the induction of chronic neuroinflammation.

## Conclusion

Sleep disturbances are common in both MCI and AD. There is a need to better evaluate the role of neuroinflammation associated with sleep disturbance in the progression of MCI and AD. There are methodological limitations of existing studies that must be acknowledged. Results are supported by a small number of studies, have small sample sizes, often do not differentiate between different subgroups of MCI (amnesiac versus non-amnesiac), and do not consistently address confounders, including several common comorbidities in older adults such as depression or OSA, which could also negatively impact sleep quality and AD biomarkers. Large scale human studies with more comprehensive and objective sleep measures are needed. Future studies should consider the important biological/metabolic signatures associated with sleep disturbance and neuroinflammation, and subsequent cognitive decline with age. From a clinical perspective, a better understanding of the associations between sleep disturbances, cognitive integrity, and brain integrity in older adults may allow for more targeted preventive strategies that promote healthy aging.

## Author Contributions

VP, S-HO, DB, NK, KR, and FO wrote and edited the manuscript.

## Conflict of Interest

DB was a consultant for Merck, Eisai, and Ferring in the last 12 months.

The remaining authors declare that the research was conducted in the absence of any commercial or financial relationships that could be construed as a potential conflict of interest.

## References

[B1] Alvarez A. Cacabelos R. Sanpedro C. Garcia-Fantini M. Aleixandre M. (2007). Serum TNF-alpha levels are increased and correlate negatively with free IGF-I in Alzheimer disease. *Neurobiol. Aging* 28 533–536. 10.1016/j.neurobiolaging.2006.02.012 16569464

[B2] Atienza M. Ziontz J. Cantero J. L. (2018). Low-grade inflammation in the relationship between sleep disruption, dysfunctional adiposity, and cognitive decline in aging. *Sleep Med. Rev.* 42 171–183. 10.1016/j.smrv.2018.08.002 30241997

[B3] Ballard C. Gauthier S. Corbett A. Brayne C. Aarsland D. Jones E. (2011). Alzheimer’s disease. *Lancet* 377 1019–1031. 10.1016/s0140-6736(10)61349-9 21371747

[B4] Benito-León J. Louis E. D. Bermejo-Pareja F. (2013). Cognitive decline in short and long sleepers: a prospective population-based study (NEDICES). *J. Psychiatr. Res.* 47 1998–2003. 10.1016/j.jpsychires.2013.09.007 24094933 PMC3839098

[B5] Blackwell T. Yaffe K. Ancoli-Israel S. Schneider J. L. Cauley J. A. Hillier T. A. (2006). Poor sleep is associated with impaired cognitive function in older women: the study of osteoporotic fractures. *J. Gerontol. Ser. A Biol. Sci. Med. Sci.* 61 405–410. 10.1093/gerona/61.4.405 16611709

[B6] Bliwise D. L. (1996). Is sleep apnea a cause of reversible dementia in old age? *J. Am. Geriatr. Soc.* 44 1407–1409. 10.1111/j.1532-5415.1996.tb01421.x 8909365

[B7] Bliwise D. L. Hughes M. McMahon P. M. Kutner N. (1995). Observed sleep/wakefulness and severity of dementia in an Alzheimer’s disease special care unit. *J. Gerontol. A Biol. Sci. Med. Sci.* 50 M303–M306. 10.1093/gerona/50a.6.m303 7583801

[B8] Bliwise D. L. Mercaldo N. D. Avidan A. Y. Boeve B. F. Greer S. A. Kukull W. A. (2011). Sleep disturbance in dementia with Lewy bodies and Alzheimer’s disease: a multicenter analysis. *Dement. Geriatr. Cogn. Disord.* 31 239–246. 10.1159/000326238 21474933 PMC3085031

[B9] Bliwise D. L. Tinklenberg J. R. Yesavage J. A. (1992). Timing of sleep and wakefulness in Alzheimer’s disease patients residing at home. *Biol. Psychiatry* 31 1163–1165. 10.1016/0006-3223(92)90162-S 1525280

[B10] Bliwise D. L. Trotti L. Wood-Siverio C. Hu W. (2019). APOE4, but not desaturation index, is associated with dementia severity in a memory clinic population. *Sleep* 42 A285–A286.

[B11] Blum-Degena D. Müller T. Kuhn W. Gerlach M. Przuntek H. Riederer P. (1995). Interleukin-1β and interleukin-6 are elevated in the cerebrospinal fluid of Alzheimer’s and de novo Parkinson’s disease patients. *Neurosci. Lett.* 202 17–20. 10.1016/0304-3940(95)12192-7 8787820

[B12] Bondareff W. Mountjoy C. Roth M. (1981). Selective loss of neurones of origin of adrenergic projection to cerebral cortex (nucleus locus coeruleus) in senile dementia. *Lancet* 317 783–784. 10.1016/s0140-6736(81)92657-x 6110985

[B13] Bondareff W. Mountjoy C. Roth M. Rossor M. Iversen L. Reynolds G. (1987). Neuronal degeneration in locus ceruleus and cortical correlates of Alzheimer disease. *Alzheimer Dis. Assoc. Disord.* 1 256–262. 10.1097/00002093-198701040-00005 3453748

[B14] Braak H. Thal D. R. Ghebremedhin E. Del Tredici K. (2011). Stages of the pathologic process in Alzheimer disease: age categories from 1 to 100 years. *J. Neuropathol. Exp. Neurol.* 70 960–969. 10.1097/nen.0b013e318232a379 22002422

[B15] Brayet P. Petit D. Frauscher B. Gagnon J. F. Gosselin N. Gagnon K. (2016). Quantitative EEG of rapid-eye-movement sleep: a marker of amnestic mild cognitive impairment. *Clin. EEG Neurosci.* 47 134–141. 10.1177/1550059415603050 26323578

[B16] Bu X. L. Liu Y. H. Wang Q. H. Jiao S. S. Zeng F. Yao X. Q. (2015). Serum amyloid-beta levels are increased in patients with obstructive sleep apnea syndrome. *Sci. Rep.* 5:13917 10.1038/srep13917 PMC4563592 26351108

[B17] Bumb J. M. Brockmann M. A. Groden C. Nolte I. (2013). Microstructural analysis of pineal volume using trueFISP imaging. *World J. Radiol.* 5 166–172. 23671752 10.4329/wjr.v5.i4.166 PMC3647208

[B18] Bumb J. M. Schilling C. Enning F. Haddad L. Paul F. Lederbogen F. (2014). Pineal gland volume in primary insomnia and healthy controls: a magnetic resonance imaging study. *J. Sleep Res.* 23 276–282. 10.1111/jsr.12125 24456088

[B19] Cacabelos R. Alvarez X. Fernandez-Novoa L. Franco A. Mangues R. Pellicer A. (1994). Brain interleukin-1 beta in Alzheimer’s disease and vascular dementia. *Methods Find. Exp. Clin. Pharmacol.* 16 141–151. 8007743

[B20] Cardona A. E. Pioro E. P. Sasse M. E. Kostenko V. Cardona S. M. Dijkstra I. M. (2006). Control of microglial neurotoxicity by the fractalkine receptor. *Nat. Neurosci.* 9 917–924. 10.1038/nn1715 16732273

[B21] Carvalho D. Z. St Louis E. K. Knopman D. S. Boeve B. F. Lowe V. J. Roberts R. O. (2018). Association of excessive daytime sleepiness with longitudinal beta-amyloid accumulation in elderly persons without dementia. *JAMA Neurol.* 75 672–680. 10.1001/jamaneurol.2018.0049 29532057 PMC5885188

[B22] Cecon E. Chen M. Marçola M. Fernandes P. A. Jockers R. Markus R. P. (2015). Amyloid β peptide directly impairs pineal gland melatonin synthesis and melatonin receptor signaling through the ERK pathway. *FASEB J.* 29 2566–2582. 10.1096/fj.14-265678 25757565

[B23] Cedernaes J. Osorio R. S. Varga A. W. Kam K. Schiöth H. B. Benedict C. (2017). Candidate mechanisms underlying the association between sleep-wake disruptions and Alzheimer’s disease. *Sleep Med. Rev.* 31 102–111. 10.1016/j.smrv.2016.02.002 26996255 PMC4981560

[B24] Cermakian N. Waddington Lamont E. Boudreau P. Boivin D. B. (2011). Circadian clock gene expression in brain regions of Alzheimer’s disease patients and control subjects. *J. Biol. Rhythms* 26 160–170. 10.1177/0748730410395732 21454296

[B25] Chang R. Yee K.-L. Sumbria R. K. (2017). Tumor necrosis factor α Inhibition for Alzheimer’s disease. *J. Cent. Nerv. Syst. Dis.* 9 1179573517709278. 10.1177/1179573517709278 PMC5436834 28579870

[B26] Chennaoui M. Sauvet F. Drogou C. Van Beers P. Langrume C. Guillard M. (2011). Effect of one night of sleep loss on changes in tumor necrosis factor alpha (TNF-α) levels in healthy men. *Cytokine* 56 318–324. 10.1016/j.cyto.2011.06.002 21737301

[B27] Cho S.-H. Sun B. Zhou Y. Kauppinen T. M. Halabisky B. Wes P. (2011). CX3CR1 protein signaling modulates microglial activation and protects against plaque-independent cognitive deficits in a mouse model of Alzheimer disease. *J. Biol. Chem.* 286 32713–32722. 10.1074/jbc.m111.254268 21771791 PMC3173153

[B28] Chong Y. (1997). Effect of a carboxy-terminal fragment of the Alzheimer’s amyloid precursor protein on expression of proinflammatory cytokines in rat glial cells. *Life Sci.* 61 2323–2333. 10.1016/s0024-3205(97)00936-3 9408055

[B29] Corder E. H. Saunders A. M. Strittmatter W. J. Schmechel D. E. Gaskell P. C. Small G. (1993). Gene dose of apolipoprotein E type 4 allele and the risk of Alzheimer’s disease in late onset families. *Science* 261 921–923. 10.1126/science.8346443 8346443

[B30] da Silva R. A. (2015). Sleep disturbances and mild cognitive impairment: a review. *Sleep Sci.* 8 36–41. 10.1016/j.slsci.2015.02.001 26483941 PMC4608881

[B31] Daulatzai M. A. (2012). Quintessential risk factors: their role in promoting cognitive dysfunction and Alzheimer’s disease. *Neurochem. Res.* 37 2627–2658. 10.1007/s11064-012-0854-6 22886562

[B32] Daulatzai M. A. (2013). Neurotoxic saboteurs: straws that break the Hippo’s (Hippocampus) back drive cognitive impairment and Alzheimer’s disease. *Neurotox. Res.* 24 407–459. 10.1007/s12640-013-9407-2 23820984

[B33] Deng Y.-Q. Xu G.-G. Duan P. Zhang Q. Wang J.-Z. (2005). Effects of melatonin on wortmannin-induced tau hyperphosphorylation. *Acta Pharmacol. Sin.* 26 519–526. 10.1111/j.1745-7254.2005.00102.x 15842767

[B34] Devore E. E. Grodstein F. Duffy J. F. Stampfer M. J. Czeisler C. A. Schernhammer E. S. (2014). Sleep duration in midlife and later life in relation to cognition. *J. Am. Geriatr. Soc.* 62 1073–1081. 10.1111/jgs.12790 24786726 PMC4188530

[B35] Diekelmann S. Born J. (2010). The memory function of sleep. *Nat. Rev. Neurosci.* 11 114–126. 20046194 10.1038/nrn2762

[B36] Drogos L. L. Gill S. J. Tyndall A. V. Raneri J. K. Parboosingh J. S. Naef A. (2016). Evidence of association between sleep quality and APOE ε4 in healthy older adults: a pilot study. *Neurology* 87 1836–1842. 10.1212/wnl.0000000000003255 27777343 PMC5089524

[B37] Eckel-Mahan K. Sassone-Corsi P. (2013). Metabolism and the circadian clock converge. *Physiol. Rev.* 93 107–135. 10.1152/physrev.00016.2012 23303907 PMC3781773

[B38] Feinstein D. L. Kalinin S. Braun D. (2016). Causes, consequences, and cures for neuroinflammation mediated via the locus coeruleus: noradrenergic signaling system. *J. Neurochem.* 139 154–178. 10.1111/jnc.13447 26968403

[B39] Frey D. J. Fleshner M. Wright K. P. Jr. (2007). The effects of 40 hours of total sleep deprivation on inflammatory markers in healthy young adults. *Brain Behav. Immun.* 21 1050–1057. 10.1016/j.bbi.2007.04.003 17524614

[B40] Gachon F. Loizides-Mangold U. Petrenko V. Dibner C. (2017). Glucose homeostasis: regulation by peripheral circadian clocks in rodents and humans. *Endocrinology* 158 1074–1084. 10.1210/en.2017-00218 28324069

[B41] Gispert J. D. Monte G. C. Falcon C. Tucholka A. Rojas S. Sanchez-Valle R. (2016). CSF YKL-40 and pTau181 are related to different cerebral morphometric patterns in early AD. *Neurobiol. Aging* 38 47–55. 10.1016/j.neurobiolaging.2015.10.022 26827642

[B42] Gottlieb D. DeStefano A. Foley D. Mignot E. Redline S. Givelber R. (2004). APOE ε4 is associated with obstructive sleep apnea/hypopnea: the Sleep Heart Health Study. *Neurology* 63 664–668. 10.1212/01.wnl.0000134671.99649.32 15326239

[B43] Guarnieri B. Adorni F. Musicco M. Appollonio I. Bonanni E. Caffarra P. (2012). Prevalence of sleep disturbances in mild cognitive impairment and dementing disorders: a multicenter Italian clinical cross-sectional study on 431 patients. *Dement. Geriatr. Cogn. Disord.* 33 50–58. 10.1159/000335363 22415141 PMC3696366

[B44] Guerreiro R. Wojtas A. Bras J. Carrasquillo M. Rogaeva E. Majounie E. (2013). TREM2 variants in Alzheimer’s disease. *N. Engl. J. Med.* 368 117–127. 23150934 10.1056/NEJMoa1211851 PMC3631573

[B45] Hansson O. Zetterberg H. Buchhave P. Londos E. Blennow K. Minthon L. (2006). Association between CSF biomarkers and incipient Alzheimer’s disease in patients with mild cognitive impairment: a follow-up study. *Lancet Neurol.* 5 228–234. 10.1016/S1474-4422(06)70355-6 16488378

[B46] Hardy J. A. Higgins G. A. (1992). Alzheimer’s disease: the amyloid cascade hypothesis. *Science* 256 184–186. 1566067 10.1126/science.1566067

[B47] Hayashi Y. Koyanagi S. Kusunose N. Okada R. Wu Z. Tozaki-Saitoh H. (2013). The intrinsic microglial molecular clock controls synaptic strength via the circadian expression of cathepsin S. *Sci. Rep.* 3:2744. 10.1038/srep02744 PMC3783043 24067868

[B48] He J. Hsuchou H. He Y. Kastin A. J. Wang Y. Pan W. (2014). Sleep restriction impairs blood-brain barrier function. *J. Neurosci.* 34 14697–14706. 10.1523/JNEUROSCI.2111-14.2014 25355222 PMC4212067

[B49] He P. Zhong Z. Lindholm K. Berning L. Lee W. Lemere C. (2007). Deletion of tumor necrosis factor death receptor inhibits amyloid β generation and prevents learning and memory deficits in Alzheimer’s mice. *J. Cell Biol.* 178 829–841. 10.1083/jcb.200705042 17724122 PMC2064547

[B50] Heneka M. T. Carson M. J. El Khoury J. Landreth G. E. Brosseron F. Feinstein D. L. (2015). Neuroinflammation in Alzheimer’s disease. *Lancet Neurol.* 14 388–405. 25792098 10.1016/S1474-4422(15)70016-5 PMC5909703

[B51] Hita-Yanez E. Atienza M. Gil-Neciga E. Cantero J. L. (2012). Disturbed sleep patterns in elders with mild cognitive impairment: the role of memory decline and ApoE epsilon4 genotype. *Curr. Alzheimer Res.* 9 290–297. 10.2174/156720512800107609 22211488

[B52] Hsu J. C. Lee Y. S. Chang C. N. Chuang H. L. Ling E. A. Lan C. T. (2003). Sleep deprivation inhibits expression of NADPH-d and NOS while activating microglia and astroglia in the rat hippocampus. *Cells Tissues Organs* 173 242–254. 10.1159/000070380 12766354

[B53] Huang Y. Potter R. Sigurdson W. Santacruz A. Shih S. Ju Y.-E. (2012). Effects of age and amyloid deposition on Aβ dynamics in the human central nervous system. *Arch. Neurol.* 69 51–58. 10.1001/archneurol.2011.235 21911660 PMC3254706

[B54] Irwin M. R. Vitiello M. V. (2019). Implications of sleep disturbance and inflammation for Alzheimer’s disease dementia. *Lancet Neurol*. 18 296–306. 10.1016/s1474-4422(18)30450-2 30661858

[B55] Jay T. R. Hirsch A. M. Broihier M. L. Miller C. M. Neilson L. E. Ransohoff R. M. (2017). Disease progression-dependent effects of TREM2 deficiency in a mouse model of Alzheimer’s disease. *J. Neurosci.* 37 637–647. 10.1523/jneurosci.2110-16.2016 28100745 PMC5242410

[B56] Jay T. R. Miller C. M. Cheng P. J. Graham L. C. Bemiller S. Broihier M. L. (2015). TREM2 deficiency eliminates TREM2+ inflammatory macrophages and ameliorates pathology in Alzheimer’s disease mouse models. *J. Exp. Med.* 212 287–295. 10.1084/jem.20142322 25732305 PMC4354365

[B57] Jessen N. A. Munk A. S. F. Lundgaard I. Nedergaard M. (2015). The glymphatic system: a beginner’s guide. *Neurochem. Res.* 40 2583–2599. 25947369 10.1007/s11064-015-1581-6 PMC4636982

[B58] Jonsson T. Stefansson H. Steinberg S. Jonsdottir I. Jonsson P. V. Snaedal J. (2013). Variant of TREM2 associated with the risk of Alzheimer’s disease. *N. Engl. J. Med.* 368 107–116. 23150908 10.1056/NEJMoa1211103 PMC3677583

[B59] Ju Y.-E. S. McLeland J. S. Toedebusch C. D. Xiong C. Fagan A. M. Duntley S. P. (2013). Sleep quality and preclinical Alzheimer disease. *JAMA Neurol.* 70 587–593. 23479184 10.1001/jamaneurol.2013.2334 PMC3676720

[B60] Ju Y.-E. S. Ooms S. J. Sutphen C. Macauley S. L. Zangrilli M. A. Jerome G. (2017). Slow wave sleep disruption increases cerebrospinal fluid amyloid-β levels. *Brain* 140 2104–2111. 10.1093/brain/awx148 28899014 PMC5790144

[B61] Jung S. Aliberti J. Graemmel P. Sunshine M. J. Kreutzberg G. W. Sher A. (2000). Analysis of fractalkine receptor CX3CR1 function by targeted deletion and green fluorescent protein reporter gene insertion. *Mol. Cell. Biol.* 20 4106–4114. 10.1128/mcb.20.11.4106-4114.2000 10805752 PMC85780

[B62] Kadotani H. Kadotani T. Young T. Peppard P. E. Finn L. Colrain I. M. (2001). Association between apolipoprotein e? 4 and sleep-disordered breathing in adults. *JAMA* 285 2888–2890. 11401610 10.1001/jama.285.22.2888

[B63] Keage H. A. D. Banks S. Yang K. L. Morgan K. Brayne C. Matthews F. E. (2012). What sleep characteristics predict cognitive decline in the elderly? *Sleep Med.* 13 886–892. 10.1016/j.sleep.2012.02.003 22560827

[B64] Killgore W. D. S. (2010). Effects of sleep deprivation on cognition. *Prog. Brain Res.* 185 105–129. 10.1016/b978-0-444-53702-7.00007-5 21075236

[B65] Kim T.-S. Lim H.-K. Lee J. Y. Kim D.-J. Park S. Lee C. (2008). Changes in the levels of plasma soluble fractalkine in patients with mild cognitive impairment and Alzheimer’s disease. *Neurosci. Lett.* 436 196–200. 10.1016/j.neulet.2008.03.019 18378084

[B66] Koo K. Y. G. Schweizer T. A. Fischer C. E. Munoz D. G. (2019). Abnormal sleep behaviours across the spectrum of Alzheimer’s disease severity: influence of Apoe genotypes and Lewy Bodies. *Curr. Alzheimer Res.* 16 243–250. 10.2174/1567205016666190103161034 30605058 PMC6613208

[B67] Krabbe G. Halle A. Matyash V. Rinnenthal J. L. Eom G. D. Bernhardt U. (2013). Functional impairment of microglia coincides with Beta-amyloid deposition in mice with Alzheimer-like pathology. *PLoS One* 8:e60921 10.1371/journal.pone.0060921 PMC3620049 23577177

[B68] Krishnaiah S. Y. Wu G. Altman B. J. Growe J. Rhoades S. D. Coldren F. (2017). Clock regulation of metabolites reveals coupling between transcription and metabolism. *Cell Metab.* 25 961–974.e4. 10.1016/j.cmet.2017.03.019 28380384 PMC5479132

[B69] Kuhn S. A. van Landeghem F. K. Zacharias R. Färber K. Rappert A. Pavlovic S. (2004). Microglia express GABAB receptors to modulate interleukin release. *Mol. Cell. Neurosci.* 25 312–322. 10.1016/j.mcn.2003.10.023 15019947

[B70] Lahiri D. K. (1999). Melatonin affects the metabolism of the β-amyloid precursor protein in different cell types. *J. Pineal Res.* 26 137–146. 10.1111/j.1600-079x.1999.tb00575.x 10231726

[B71] Lee J. H. Bliwise D. L. Ansari F. P. Goldstein F. C. Cellar J. S. Lah J. J. (2007). Daytime sleepiness and functional impairment in Alzheimer disease. *Am. J. Geriatr. Psychiatry* 15 620–626. 10.1097/JGP.0b013e3180381521 17586786

[B72] Lee M. Schwab C. Mcgeer P. L. (2011). Astrocytes are GABAergic cells that modulate microglial activity. *Glia* 59 152–165. 10.1002/glia.21087 21046567

[B73] Li R. Yang L. Lindholm K. Konishi Y. Yue X. Hampel H. (2004). Tumor necrosis factor death receptor signaling cascade is required for amyloid-β protein-induced neuron death. *J. Neurosci.* 24 1760–1771. 10.1523/jneurosci.4580-03.2004 14973251 PMC6730458

[B74] Liao Y.-F. Wang B.-J. Cheng H.-T. Kuo L.-H. Wolfe M. S. (2004). Tumor necrosis factor-α, interleukin-1β, and interferon-γ stimulate γ-secretase-mediated cleavage of amyloid precursor protein through a JNK-dependent MAPK pathway. *J. Biol. Chem.* 279 49523–49532. 10.1074/jbc.m402034200 15347683

[B75] Limatola C. Ransohoff R. M. (2014). Modulating neurotoxicity through CX3CL1/CX3CR1 signaling. *Front. Cell. Neurosci.* 8:229 10.3389/fncel.2014.00229 PMC4126442 25152714

[B76] Lotufo C. M. Yamashita C. E. Farsky S. H. Markus R. P. (2006). Melatonin effect on endothelial cells reduces vascular permeability increase induced by leukotriene B4. *Eur. J. Pharmacol.* 534 258–263. 10.1016/j.ejphar.2006.01.050 16612844

[B77] Maestri M. Carnicelli L. Tognoni G. Di Coscio E. Giorgi F. S. Volpi L. (2015). Non-rapid eye movement sleep instability in mild cognitive impairment: a pilot study. *Sleep Med.* 16 1139–1145. 10.1016/j.sleep.2015.04.027 26298791

[B78] Mander B. A. Winer J. R. Jagust W. J. Walker M. P. (2016). Sleep: a novel mechanistic pathway, biomarker, and treatment target in the pathology of Alzheimer’s disease? *Trends Neurosci.* 39 552–566. 10.1016/j.tins.2016.05.002 27325209 PMC4967375

[B79] Matsuoka T. Imai A. Fujimoto H. Kato Y. Shibata K. Nakamura K. (2017). Reduced pineal volume in alzheimer disease: a retrospective cross-sectional MR imaging study. *Radiology* 286 239–248. 10.1148/radiol.2017170188 28745939

[B80] McGeer E. G. McGeer P. L. (2010). Neuroinflammation in Alzheimer’s disease and mild cognitive impairment: a field in its infancy. *J. Alzheimers Dis.* 19 355–361. 10.3233/jad-2010-1219 20061650

[B81] McKhann G. Drachman D. Folstein M. Katzman R. Price D. Stadlan E. M. (1984). Clinical diagnosis of Alzheimer’s disease: Report of the NINCDS-ADRDA Work Group^∗^ under the auspices of Department of Health and Human Services Task Force on Alzheimer’s Disease. *Neurology* 34 939–939. 10.1212/wnl.34.7.939 6610841

[B82] Miguel-Álvarez M. Santos-Lozano A. Sanchis-Gomar F. Fiuza-Luces C. Pareja-Galeano H. Garatachea N. (2015). Non-steroidal anti-inflammatory drugs as a treatment for Alzheimer’s disease: a systematic review and meta-analysis of treatment effect. *Drugs Aging* 32 139–147. 10.1007/s40266-015-0239-z 25644018

[B83] Mizuno T. Kawanokuchi J. Numata K. Suzumura A. (2003). Production and neuroprotective functions of fractalkine in the central nervous system. *Brain Res.* 979 65–70. 10.1016/s0006-8993(03)02867-1 12850572

[B84] Montagne A. Barnes S. R. Sweeney M. D. Halliday M. R. Sagare A. P. Zhao Z. (2015). Blood-brain barrier breakdown in the aging human hippocampus. *Neuron* 85 296–302. 10.1016/j.neuron.2014.12.032 25611508 PMC4350773

[B85] Montplaisir J. Petit D. Gauthier S. Gaudreau H. Decary A. (1998). Sleep disturbances and eeg slowing in Alzheimer’s disease. *Sleep Res. Online* 1 147–151. 11382871

[B86] Muangpaisan W. Intalapaporn S. Assantachai P. (2008). Neuropsychiatric symptoms in the community-based patients with mild cognitive impairment and the influence of demographic factors. *Int. J. Geriatr. Psychiatry* 23 699–703. 10.1002/gps.1963 18172914

[B87] Mullington J. M. Abbott S. M. Carroll J. E. Davis C. J. Dijk D.-J. Dinges D. F. (2016). Developing biomarker arrays predicting sleep and circadian-coupled risks to health. *Sleep* 39 727–736. 10.5665/sleep.5616 26951388 PMC4791606

[B88] Musiek E. S. (2017). Circadian rhythms in AD pathogenesis: a critical appraisal. *Curr. Sleep Med. Rep.* 3 85–92. 10.1007/s40675-017-0072-5 29308355 PMC5754029

[B89] Naismith S. L. Rogers N. L. Hickie I. B. Mackenzie J. Norrie L. M. Lewis S. J. (2010). Sleep well, think well: sleep-wake disturbance in mild cognitive impairment. *J. Geriatr. Psychiatry Neurol.* 23 123–130. 10.1177/0891988710363710 20354239

[B90] Nayak D. Roth T. L. McGavern D. B. (2014). Microglia development and function. *Annu. Rev. Immunol.* 32 367–402. 10.1146/annurev-immunol-032713-120240 24471431 PMC5001846

[B91] Ni J. Wu Z. Meng J. Saito T. Saido T. C. Qing H. (2019). An impaired intrinsic microglial clock system induces neuroinflammatory alterations in the early stage of amyloid precursor protein knock-in mouse brain. *J. Neuroinflammation* 16:173. 10.1186/s12974-019-1562-9 PMC6716829 31470863

[B92] Ojo J. O. Rezaie P. Gabbott P. L. Stewart M. G. (2015). Impact of age-related neuroglial cell responses on hippocampal deterioration. *Front. Aging Neurosci.* 7:57 10.3389/fnagi.2015.00057 PMC4413780 25972808

[B93] Okun M. Giese S. Lin L. Einen M. Mignot E. Coussons-Read M. (2004). Exploring the cytokine and endocrine involvement in narcolepsy. *Brain Behav. Immun.* 18 326–332. 10.1016/j.bbi.2003.11.002 15157949

[B94] Olsson B. Hertze J. Lautner R. Zetterberg H. Nagga K. Hoglund K. (2013). Microglial markers are elevated in the prodromal phase of Alzheimer’s disease and vascular dementia. *J. Alzheimers Dis.* 33 45–53. 10.3233/jad-2012-120787 22890100

[B95] Olsson M. Ärlig J. Hedner J. Blennow K. Zetterberg H. (2018). Sleep deprivation and cerebrospinal fluid biomarkers for Alzheimer’s disease. *Sleep* 41:zsy025 10.1093/sleep/zsy025 29425372

[B96] Ooms S. Overeem S. Besse K. Rikkert M. O. Verbeek M. Claassen J. A. (2014). Effect of 1 night of total sleep deprivation on cerebrospinal fluid beta-amyloid 42 in healthy middle-aged men: a randomized clinical trial. *JAMA Neurol.* 71 971–977. 10.1001/jamaneurol.2014.1173 24887018

[B97] Park M. Shah R. C. Fogg L. F. Wyatt J. K. (2011). Daytime sleepiness in mild Alzheimer’s disease with and without parkinsonian features. *Sleep Med.* 12 397–402. 10.1016/j.sleep.2010.09.006 21388877

[B98] Petersen R. C. Smith G. E. Waring S. C. Ivnik R. J. Tangalos E. G. Kokmen E. (1999). Mild cognitive impairment: clinical characterization and outcome. *Arch. Neurol.* 56 303–308. 10190820 10.1001/archneur.56.3.303

[B99] Potvin O. Lorrain D. Forget H. Dubé M. Grenier S. Préville M. (2012). Sleep quality and 1-year incident cognitive impairment in community-dwelling older adults. *Sleep* 35 491–499. 10.5665/sleep.1732 22467987 PMC3296791

[B100] Reitz C. Mayeux R. (2014). Alzheimer disease: epidemiology, diagnostic criteria, risk factors and biomarkers. *Biochem. Pharmacol.* 88 640–651. 10.1016/j.bcp.2013.12.024 24398425 PMC3992261

[B101] Ringheim G. E. Szczepanik A. M. Petko W. Burgher K. L. Zu Zhu S. Chao C. C. (1998). Enhancement of beta-amyloid precursor protein transcription and expression by the soluble interleukin-6 receptor/interleukin-6 complex. *Mol. Brain Res.* 55 35–44. 10.1016/s0169-328x(97)00356-2 9645958

[B102] Robinson D. (1975). Changes in monoamine oxidase and monoamines with human development and aging. *Fed. Proc.* 34 103–107. 1088942

[B103] Rosenzweig I. Glasser M. Crum W. R. Kempton M. J. Milosevic M. McMillan A. (2016). Changes in neurocognitive architecture in patients with obstructive sleep apnea treated with continuous positive airway pressure. *EBioMedicine* 7 221–229. 10.1016/j.ebiom.2016.03.020 27322475 PMC4909326

[B104] Rothaug M. Becker-Pauly C. Rose-John S. (2016). The role of interleukin-6 signaling in nervous tissue. *Biochim. Biophys. Acta* 1863 1218–1227. 10.1016/j.bbamcr.2016.03.018 27016501

[B105] Savage J. C. Jay T. Goduni E. Quigley C. Mariani M. M. Malm T. (2015). Nuclear receptors license phagocytosis by trem2+ myeloid cells in mouse models of Alzheimer’s disease. *J. Neurosci.* 35 6532–6543. 10.1523/jneurosci.4586-14.2015 25904803 PMC4405560

[B106] Savaskan E. Ayoub M. A. Ravid R. Angeloni D. Fraschini F. Meier F. (2005). Reduced hippocampal MT2 melatonin receptor expression in Alzheimer’s disease. *J. Pineal Res.* 38 10–16. 10.1111/j.1600-079x.2004.00169.x 15617532

[B107] Savaskan E. Olivieri G. Meier F. Brydon L. Jockers R. Ravid R. (2002). Increased melatonin 1a-receptor immunoreactivity in the hippocampus of Alzheimer’s disease patients. *J. Pineal Res.* 32 59–62. 10.1034/j.1600-079x.2002.00841.x 11841602

[B108] Schoonenboom S. Visser P. J. Mulder C. Lindeboom J. Van Elk E.-J. Van Kamp G. J. (2005). Biomarker profiles and their relation to clinical variables in mild cognitive impairment. *Neurocase* 11 8–13. 10.1080/13554790490896785 15804919

[B109] Schuitemaker A. Dik M. G. Veerhuis R. Scheltens P. Schoonenboom N. S. Hack C. E. (2009). Inflammatory markers in AD and MCI patients with different biomarker profiles. *Neurobiol. Aging* 30 1885–1889. 10.1016/j.neurobiolaging.2008.01.014 18378357

[B110] Sharma R. A. Varga A. W. Bubu O. M. Pirraglia E. Kam K. Parekh A. (2018). Obstructive sleep apnea severity affects amyloid burden in cognitively normal elderly. A longitudinal study. *Am. J. Respir. Crit. Care Med.* 197 933–943. 29125327 10.1164/rccm.201704-0704OC PMC6020410

[B111] Shokri-Kojori E. Wang G.-J. Wiers C. E. Demiral S. B. Guo M. Kim S. W. (2018). β-Amyloid accumulation in the human brain after one night of sleep deprivation. *Proc. Natl. Acad. Sci. U.S.A.* 115 4483–4488. 10.1073/pnas.1721694115 29632177 PMC5924922

[B112] Singh-Manoux A. Dugravot A. Brunner E. Kumari M. Shipley M. Elbaz A. (2014). Interleukin-6 and C-reactive protein as predictors of cognitive decline in late midlife. *Neurology* 83 486–493. 10.1212/wnl.0000000000000665 24991031 PMC4141998

[B113] Spira A. P. An Y. Wu M. N. Owusu J. T. Simonsick E. M. Bilgel M. (2018). Excessive daytime sleepiness and napping in cognitively normal adults: associations with subsequent amyloid deposition measured by PiB PET. *Sleep* 41:zsy152 10.1093/sleep/zsy152 PMC6289234 30257013

[B114] Spira A. P. Gamaldo A. A. An Y. Wu M. N. Simonsick E. M. Bilgel M. (2013). Self-reported Sleep and β-Amyloid Deposition in Community-Dwelling Older AdultsSelf-reported Sleep and β-Amyloid DepositionSelf-reported Sleep and β-Amyloid Deposition. *JAMA Neurol.* 70 1537–1543. 10.1001/jamaneurol.2013.4258 24145859 PMC3918480

[B115] Spira A. P. Yager C. Brandt J. Smith G. S. Zhou Y. Mathur A. (2014). Objectively measured sleep and beta-amyloid burden in older adults: a pilot study. *SAGE Open Med.* 2:2050312114546520 10.1177/2050312114546520 PMC4304392 25621174

[B116] Sprecher K. E. Bendlin B. B. Racine A. M. Okonkwo O. C. Christian B. T. Koscik R. L. (2015). Amyloid burden is associated with self-reported sleep in nondemented late middle-aged adults. *Neurobiol. Aging* 36 2568–2576. 10.1016/j.neurobiolaging.2015.05.004 26059712 PMC4523445

[B117] Sprecher K. E. Koscik R. L. Carlsson C. M. Zetterberg H. Blennow K. Okonkwo O. C. (2017). Poor sleep is associated with CSF biomarkers of amyloid pathology in cognitively normal adults. *Neurology* 89 445–453. 10.1212/wnl.0000000000004171 28679595 PMC5539733

[B118] Tamura E. K. Cecon E. Monteiro A. W. A. Silva C. L. M. Markus R. P. (2009). Melatonin inhibits LPS-induced NO production in rat endothelial cells. *J. Pineal Res.* 46 268–274. 10.1111/j.1600-079x.2008.00657.x 19215575

[B119] Tsapanou A. Gu Y. Manly J. Schupf N. Tang M.-X. Zimmerman M. (2015). Daytime sleepiness and sleep inadequacy as risk factors for dementia. *Dement. Geriatr. Cogn. Disord. Extra* 5 286–295. 10.1159/000431311 PMC4521063 26273244

[B120] Twigg G. L. Papaioannou I. Jackson M. Ghiassi R. Shaikh Z. Jaye J. (2010). Obstructive sleep apnea syndrome is associated with deficits in verbal but not visual memory. *Am. J. Respir. Crit. Care Med.* 182 98–103. 10.1164/rccm.200901-0065oc 20299536 PMC2902762

[B121] Tworoger S. S. Lee S. Schernhammer E. S. Grodstein F. (2006). The association of self-reported sleep duration, difficulty sleeping, and snoring with cognitive function in older women. *Alzheimer Dis. Assoc. Disord.* 20 41–48. 10.1097/01.wad.0000201850.52707.80 16493235

[B122] Vgontzas A. N. Papanicolaou D. A. Bixler E. O. Kales A. Tyson K. Chrousos G. P. (1997). Elevation of plasma cytokines in disorders of excessive daytime sleepiness: role of sleep disturbance and obesity. *J. Clin. Endocrinol. Metab.* 82 1313–1316. 10.1210/jcem.82.5.3950 9141509

[B123] Vgontzas A. N. Zoumakis E. Bixler E. O. Lin H.-M. Follett H. Kales A. (2004). Adverse effects of modest sleep restriction on sleepiness, performance, and inflammatory cytokines. *J. Clin. Endocrinol. Metab.* 89 2119–2126. 10.1210/jc.2003-031562 15126529

[B124] Vitiello M. V. Prinz P. N. Williams D. E. Frommlet M. S. Ries R. K. (1990). Sleep disturbances in patients with mild-stage Alzheimer’s disease. *J. Gerontol.* 45 M131–M138. 2365965 10.1093/geronj/45.4.m131

[B125] Wang Y. Cella M. Mallinson K. Ulrich J. D. Young K. L. Robinette M. L. (2015). TREM2 lipid sensing sustains the microglial response in an Alzheimer’s disease model. *Cell* 160 1061–1071. 10.1016/j.cell.2015.01.049 25728668 PMC4477963

[B126] Weissova K. Bartoš A. Sládek M. Novakova M. Sumová A. (2016). Moderate changes in the circadian system of Alzheimer’s disease patients detected in their home environment. *PLoS One* 11:e0146200 10.1371/journal.pone.0146200 PMC4701009 26727258

[B127] Westerberg C. E. Mander B. A. Florczak S. M. Weintraub S. Mesulam M. M. Zee P. C. (2012). Concurrent impairments in sleep and memory in amnestic mild cognitive impairment. *J. Int. Neuropsychol. Soc.* 18 490–500. 10.1017/s135561771200001x 22300710 PMC3468412

[B128] Wisor J. P. Schmidt M. A. Clegern W. C. (2011). Cerebral microglia mediate sleep/wake and neuroinflammatory effects of methamphetamine. *Brain Behav. Immun.* 25 767–776. 10.1016/j.bbi.2011.02.002 21333736

[B129] Wu Y. H. Swaab D. F. (2005). The human pineal gland and melatonin in aging and Alzheimer’s disease. *J. Pineal Res.* 38 145–152. 10.1111/j.1600-079x.2004.00196.x 15725334

[B130] Xie L. Kang H. Xu Q. Chen M. J. Liao Y. Thiyagarajan M. (2013). Sleep drives metabolite clearance from the adult brain. *Science* 342 373–377. 10.1126/science.1241224 24136970 PMC3880190

[B131] Xie Y. Ba L. Wang M. Deng S. Y. Chen S. M. Huang L. F. (2019). Chronic sleep fragmentation shares similar pathogenesis with neurodegenerative diseases: Endosome-autophagosome-lysosome pathway dysfunction and microglia-mediated neuroinflammation. *CNS Neurosci. Ther.* 26 215–227. 10.1111/cns.13218 31549780 PMC6978272

[B132] Xu L. Jiang C. Q. Lam T. H. Liu B. Jin Y. L. Zhu T. (2011). Short or long sleep duration is associated with memory impairment in older Chinese: the Guangzhou Biobank Cohort Study. *Sleep* 34 575–580. 10.1093/sleep/34.5.575 21532950 PMC3079936

[B133] Yaffe K. Falvey C. M. Hoang T. (2014). Connections between sleep and cognition in older adults. *Lancet Neurol.* 13 1017–1028. 10.1016/S1474-4422(14)70172-3 25231524

[B134] Yaffe K. Laffan A. M. Harrison S. L. Redline S. Spira A. P. Ensrud K. E. (2011). Sleep-disordered breathing, hypoxia, and risk of mild cognitive impairment and dementia in older women. *JAMA* 306 613–619. 21828324 10.1001/jama.2011.1115 PMC3600944

[B135] You J. C. Jones E. Cross D. E. Lyon A. C. Kang H. Newberg A. B. (2019). Association of β-amyloid burden with sleep dysfunction and cognitive impairment in elderly individuals with cognitive disorders. *JAMA Netw. Open* 2:e1913383 10.1001/jamanetworkopen.2019.13383 PMC6806437 31617927

[B136] Yun C.-H. Lee H.-Y. Lee S. K. Kim H. Seo H. S. Bang S. (2017). Amyloid burden in obstructive sleep apnea. *J. Alzheimers Dis.* 59 21–29. 28550245 10.3233/JAD-161047

[B137] Zhou J. N. Liu R. Y. Kamphorst W. Hofman M. A. Swaab D. F. (2003). Early neuropathological Alzheimer’s changes in aged individuals are accompanied by decreased cerebrospinal fluid melatonin levels. *J. Pineal Res.* 35 125–130. 10.1034/j.1600-079x.2003.00065.x 12887656

[B138] Zhu B. Dong Y. Xu Z. Gompf H. S. Ward S. A. Xue Z. (2012). Sleep disturbance induces neuroinflammation and impairment of learning and memory. *Neurobiol. Dis.* 48 348–355. 10.1016/j.nbd.2012.06.022 22776332 PMC3461115

[B139] Zujovic V. Benavides J. Vigé X. Carter C. Taupin V. (2000). Fractalkine modulates TNF-α secretion and neurotoxicity induced by microglial activation. *Glia* 29 305–315. 10.1002/(sici)1098-1136(20000215)29:4<305::aid-glia2>3.0.co;2-v 10652441

